# Quantification of indocyanine green near-infrared fluorescence bowel perfusion assessment in colorectal surgery

**DOI:** 10.1007/s00464-023-10140-8

**Published:** 2023-06-07

**Authors:** Robin A. Faber, Floris P. Tange, Hidde A. Galema, Thomas C. Zwaan, Fabian A. Holman, Koen C. M. J. Peeters, Pieter J. Tanis, Cornelis Verhoef, Jacobus Burggraaf, J. Sven D. Mieog, Merlijn Hutteman, Stijn Keereweer, Alexander L. Vahrmeijer, Joost R. van der Vorst, Denise E. Hilling

**Affiliations:** 1 Department of Surgery, Leiden University Medical Center, Albinusdreef 2, 2333 ZA Leiden, The Netherlands; 2 Department of Surgical Oncology and Gastrointestinal Surgery, Erasmus MC Cancer Institute, Doctor Molewaterplein 40, 3015 GD Rotterdam, The Netherlands; 3 Department of Otorhinolaryngology and Head and Neck Surgery, Erasmus MC Cancer Institute, Doctor Molewaterplein 40, 3015 GD Rotterdam, The Netherlands; 4 Centre of Human Drug Research, Zernikedreef 8, 2333 CL Leiden, The Netherlands; 5 Department of Surgery, Radboud University Medical Center, Geert Grooteplein Zuid 10, 6525 GA Nijmegen, The Netherlands

**Keywords:** Colorectal surgery, Indocyanine green, Quantification, Fluorescence imaging, Bowel perfusion

## Abstract

**Background:**

Indocyanine green near-infrared fluorescence bowel perfusion assessment has shown its potential benefit in preventing anastomotic leakage. However, the surgeon's subjective visual interpretation of the fluorescence signal limits the validity and reproducibility of the technique. Therefore, this study aimed to identify objective quantified bowel perfusion patterns in patients undergoing colorectal surgery using a standardized imaging protocol.

**Method:**

A standardized fluorescence video was recorded. Postoperatively, the fluorescence videos were quantified by drawing contiguous region of interests (ROIs) on the bowel. For each ROI, a time-intensity curve was plotted from which perfusion parameters (*n* = 10) were derived and analyzed. Furthermore, the inter-observer agreement of the surgeon’s subjective interpretation of the fluorescence signal was assessed.

**Results:**

Twenty patients who underwent colorectal surgery were included in the study. Based on the quantified time-intensity curves, three different perfusion patterns were identified. Similar for both the ileum and colon, perfusion pattern 1 had a steep inflow that reached its peak fluorescence intensity rapidly, followed by a steep outflow. Perfusion pattern 2 had a relatively flat outflow slope immediately followed by its plateau phase. Perfusion pattern 3 only reached its peak fluorescence intensity after 3 min with a slow inflow gradient preceding it. The inter-observer agreement was poor-moderate (Intraclass Correlation Coefficient (ICC): 0.378, 95% CI 0.210–0.579).

**Conclusion:**

This study showed that quantification of bowel perfusion is a feasible method to differentiate between different perfusion patterns. In addition, the poor-moderate inter-observer agreement of the subjective interpretation of the fluorescence signal between surgeons emphasizes the need for objective quantification.

**Supplementary Information:**

The online version contains supplementary material available at 10.1007/s00464-023-10140-8.

Anastomotic leakage (AL) is a serious postoperative complication in colorectal surgery with an incidence ranging from 1 to 20% [1]. It is associated with high morbidity and mortality, prolonged hospitalization, increased healthcare costs, and impaired oncological outcomes [2, 3]. The etiology of AL is multifactorial, in which compromised bowel perfusion is considered as a major contributing factor. Conventionally, bowel perfusion is assessed intraoperatively based on subjective clinical indicators, including tissue color, peristaltic movements, active bleeding from marginal arteries and palpable mesenteric arterial pulsations. Additional tests that may be used to assess the integrity of the anastomosis are the air leak test, intraoperative endoscopy and doughnut inspection [4]. However, the surgeon’s judgement of these clinical indicators was found to have low predictive value for AL, which indicates the need for more accurate intraoperative diagnostic tests [5, 6].

Near-infrared (NIR) fluorescence imaging with indocyanine green (ICG) is a technique that enables real-time assessment of bowel perfusion. Several studies have shown its benefit to prevent AL [7]. This is currently being validated in ongoing phase III randomized controlled trials, such as the AVOID trial [8] (NCT04712032), IntAct trial [9], and EssentiAL trial (jRCTs031180039), that should determine its clinical efficacy. However, the use of this technique is based on subjective visual interpretation of the fluorescence signal by the surgeon, which remains a limiting factor for the validity and reproducibility of these data in daily practice [10–13].

Quantitative evaluation of the fluorescence signal could increase the objectivity and accuracy of ICG NIR fluorescence bowel perfusion assessment. This method is based on the analysis of fluorescence intensity over time from which various inflow and outflow parameters can be derived. Some cohort studies have already investigated quantified bowel perfusion assessment using various generic quantification software [14–25]. However, no consensus has been reached on which perfusion pattern and/or quantitative parameters can considered to be sufficiently reliable to assess bowel perfusion [26]. This is partly due to the lack of a standardized imaging protocol, given the fact that intensity-based parameters are altered by camera-to-target distance, angle of camera-to-target tissue, type of imaging system and its settings [27].

This prospective cohort study aimed to identify quantified bowel perfusion patterns in patients undergoing colorectal surgery using a standardized imaging protocol. In addition, the inter-observer agreement of the surgeon’s subjective interpretation of the fluorescence signal was assessed.

## Methods

### Study design and population

This prospective, dual center, exploratory study was conducted at the Leiden University Medical Center (LUMC) and Erasmus MC Cancer institute (EMC), according to the declaration of Helsinki (10th version, Fortaleza, 2013). Twenty patients with colorectal cancer who underwent ICG NIR fluorescence-guided colorectal resection with a primary anastomosis were included in the study.

Medical ethical approval from the ethic committee Leiden-Den Haag-Delft was obtained (MEC-2021-0876). Informed consent was given by all patients.

### Surgical procedure

A standardized imaging protocol was used to obtain fluorescence videos for quantification of the fluorescence signal. Fluorescence videos were recorded using the Quest Spectrum 2.0 camera system (Quest Medical Imaging, Middenmeer, the Netherlands). ICG NIR fluorescence bowel perfusion assessment was performed extracorporeally in all patients regardless the surgical approach chosen (i.e., open or minimally invasive surgery). In patients undergoing minimally invasive surgery (i.e., laparoscopic or robotic), the ileum and/or afferent/efferent colon was extracted extracorporeally through a Pfannenstiel incision. Before extracorporeal ICG NIR fluorescence bowel perfusion assessment, the camera was fixed in the camera arm and positioned 30 cm above the target tissue at an angle of 90 degrees. Standardized camera settings (GAIN: color 3.5 dB, ICG-fluor 20.0 dB; EXPOSURE: color 11.0 ms, ICG-fluor 50.0 ms) were maintained. After dissection of the vascular branch and prior to bowel transection, all patients received 5 mg ICG (2.5 mg/ml, Verdye, Diagnostic Green, Aschheim, Germany) intravenously followed by 10 mL saline flush according to standard care. Surgeons were allowed to change the surgical plan intraoperatively by performing an additional bowel resection based on their subjective interpretation of the fluorescence signal. Immediately after administration, a fluorescence video was recorded for 5 min. On each fluorescence video, the resection line was marked by the surgeon.

### Quantitative analyses

**Fig. 1:**
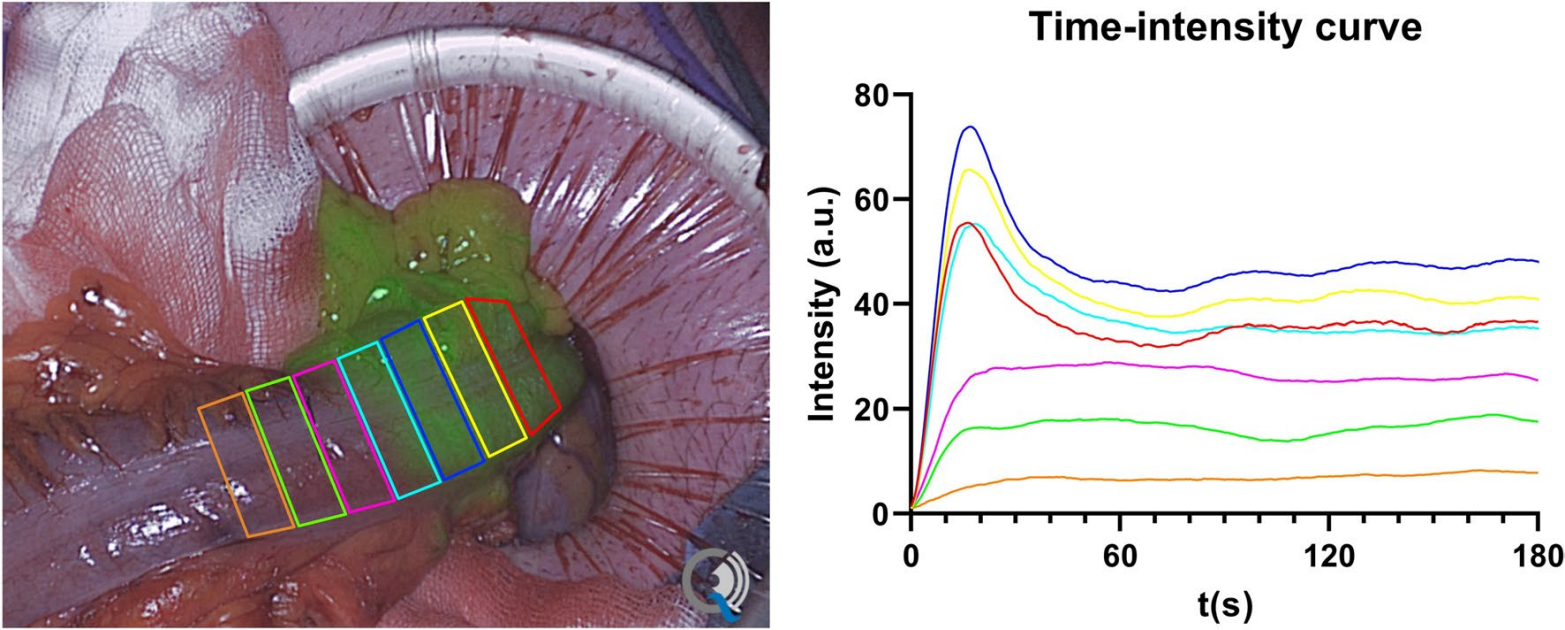
Quantification of a standardized fluorescence video using the Quest Research Framework quantification tool. Contiguous region of interests (ROIs) were drawn from proximal to distal of the resection line (located at the *dark blue* ROI) on the afferent colon. For each ROI, an absolute time-intensity curve was plotted. The color of each time-intensity curve corresponds to the color of the ROI

The Quest Research Framework (Quest Medical Imaging, Middenmeer, the Netherlands) was used for quantification of the fluorescence videos. Postoperatively, contiguous region of interests (ROIs; approximately of 1 cm length) from proximal to distal of the resection line were drawn on the ileum or afferent/efferent colon, depending on the surgical procedure performed (Fig. 1). An example of a standardized fluorescence measurement is displayed in supplementary Video 1. The fluorescence signal as shown in Video 1 was quantified from multiple ROI’s into corresponding time-intensity curves as illustrated in Fig. 1. For each ROI, absolute (i.e., fluorescence intensity over time) and normalized (i.e., fluorescence intensity as a percentual change over time by setting the maximum fluorescence intensity at 100 percent to minimize the influences of patient and camera-related factors [28]) time-intensity curves were plotted from which 5 inflow and 5 outflow parameters were derived. These time-intensity curves were analyzed separately for the ileum and colon. The inflow parameters included the maximum fluorescence intensity (Imax), maximum inflow slope (Ingress slope), mean slope from baseline to maximum fluorescence intensity (Ingress rate), maximum inflow slope in percentage per second (Normalized slope), and time to maximum intensity (Tmax); the outflow parameters included the maximum outflow slope (Egress slope), area under the curve in percentage after 30, 60, 120, and 180 s from Tmax (AUC30, AUC60, AUC120, AUC180).

### Outcomes

The primary outcome of this study was to identify quantified bowel perfusion patterns in patients undergoing colorectal surgery by analyzing the time-intensity curves plotted from each ROI and its derived inflow and outflow perfusion parameters. The secondary outcomes were to assess the inter-observer agreement of the surgeon’s subjective interpretation of the fluorescence signal and ALs after 90 days (i.e., early and late AL). The colorectal surgeons (FH, KP, DH, AB, GF, PT, CV) all experienced with fluorescence-guided surgery, were individually asked to mark the intended resection line on all 20 fluorescence videos, based on their subjective interpretation of the fluorescence signal.

The distance between the markers (i.e., intended resection lines) on each fluorescence video was measured with ImageJ (National Institutes of Health, USA), in which the most proximal marker was used as the baseline measurement according to the previously published method of Hardy et al. [12, 13]. AL was defined as AL that required active therapeutic intervention but manageable without reoperation (Grade B) or AL that requires reoperation (Grade C), according to the definition of the International Study Group of Rectal Cancer [29, 30]. To associate the quantified perfusion patterns with the occurrence of AL, the time-intensity curves plotted from the ROIs of the anastomosis were analyzed.

The time-intensity curves of the anastomosis from both the afferent ileum or colon and the efferent colon were analyzed when both could be imaged extracorporeally. If not, only the time-intensity curves of the afferent ileum or colon were analyzed.

### Statistical analyses

Patient characteristics were described using summary statistics. The distribution of all variables was assessed using histograms and verified with the Shapiro–Wilk test. Normally distributed variables were reported as means and standard deviation (SD) and skewed continuous variables were reported as medians and range. The inflow and outflow parameters from different perfusion patterns were compared using the One-way ANOVA or Kruskal Wallis test, depending on the distribution. The inter-observer agreement of the surgeon’s subjective interpretation of the fluorescence signal was assessed by calculating the intraclass correlation coefficient (ICC) according to a two-way mixed model, consistency type, single measurement method. An ICC < 0.50 indicated “poor agreement”, ICC ≥ 0.50 up to 0.75 indicated “moderate agreement”, ICC > 0.75 up to 0.90 indicated “good agreement”, and ICC > 0.90 indicated “excellent agreement”.

All statistical analyses were performed using SPSS Version 25.0. The statistical outcomes were considered significant if the *p* value was < 0.05.

## Results

### Patient characteristics

Twenty patients (13 males; 7 females) with a median age of 63 years (range 46–83) were included in the analyses. Of these patients, 7 (35%) underwent a right-sided resection (i.e., ileocecal resection, right hemicolectomy or subtotal colectomy) and 13 (65%) patients underwent a left-sided resection (i.e., left hemicolectomy, sigmoid resection or low anterior resection). The majority (85%) of patients underwent surgery for a malignant tumor. Two (10%) patients received neo-adjuvant radiotherapy prior to surgical resection. A minimally invasive approach (i.e., laparoscopic or robotic) was performed in most patients (80%). The median in-hospital stay was 5 days (range 3–90). In total, 2 patients died during hospitalization of which 1 died due to an acute coagulation disorder and 1 patient died from an acute myocardial infarction. A detailed overview of all patient characteristics is shown in Table 1.

**Table 1:** Patient characteristics

	TOTAL (*n* = 20)
Age, median (range)	63 (46–83)
Male, *n* (%)	13 (65%)
BMI^a^, *n* (%)	
< 30	14 (70%)
≥ 30	6 (30%)
ASA-score, *n* (%)	
1	1 (5%)
2	14 (70%)
3	5 (25%)
Smoking, *n* (%)	
Current	4 (20%)
Former	6 (30%)
Never	7 (35%)
Neoplasm, *n* (%)	
Benign	3 (15%)
Malignant	17 (85%)
Neo-adjuvant radiotherapy, *n* (%)	2 (10%)
Type of surgery, *n* (%)	
Right-sided resection^b^	7 (35%)
Left-sided resection^c^	13 (65%)
Surgical approach, *n* (%)	
Open	4 (20%)
Laparoscopic	11 (55%)
Robotic	5 (25%)
Type of anastomosis, *n* (%)	
Side-to-side isoperistaltic	4 (20%)
Side-to-side antiperistaltic	4 (20%)
Side-to-end	10 (50%)
End-to-side	0 (0%)
End-to-end	1 (5%)
Anastomosis technique, *n* (%)	
Handsewn	3 (15%)
Stapled	16 (80%)
Hospitalization in days, median (range)	5 (3–90)
Anastomotic leakage, *n* (%)	4 (20%)
Mortality, *n* (%)	2 (10%)

^a^Body Mass Index (weight per kilogram/length^2^)

^b^Included ileocecal resection, right hemicolectomy, and subtotal colectomy

^c^Included left hemicolectomy, sigmoid resection, and low anterior resection

### Perfusion patterns

A total of 218 ROIs were drawn on the ileum or afferent/efferent colon, with a mean number of 11 ± 3.8 ROIs per patient. From these ROIs, 35 (16%) time-intensity curves of the ileum and 183 (84%) time-intensity curves of the colon were plotted and analyzed.

Based on qualitative assessment of the quantified time-intensity curves, three different perfusion patterns were identified. Similar for both the ileum and colon, perfusion pattern 1 was characterized by a steep inflow slope (Ingress slope) that reached its peak fluorescence intensity (Tmax) rapidly, followed by a steep outflow slope (Egress slope). In contrast, perfusion pattern 2 had a relatively flat outflow slope immediately followed by its plateau phase. Perfusion pattern 3 only reached its peak fluorescence intensity after 3 min with a slow inflow gradient preceding it.

Cut-off points were determined for each perfusion pattern based on the quantified perfusion parameters. For the ileum, the time-intensity curves were distributed among the three different perfusion patterns using the following cut-off values; pattern 1: Tmax < 70 and Egress slope < − 2.5; pattern 2: Tmax < 70 and Egress slope ≥ − 2.5; pattern 3: Tmax ≥ 70.

**Fig. 2:**
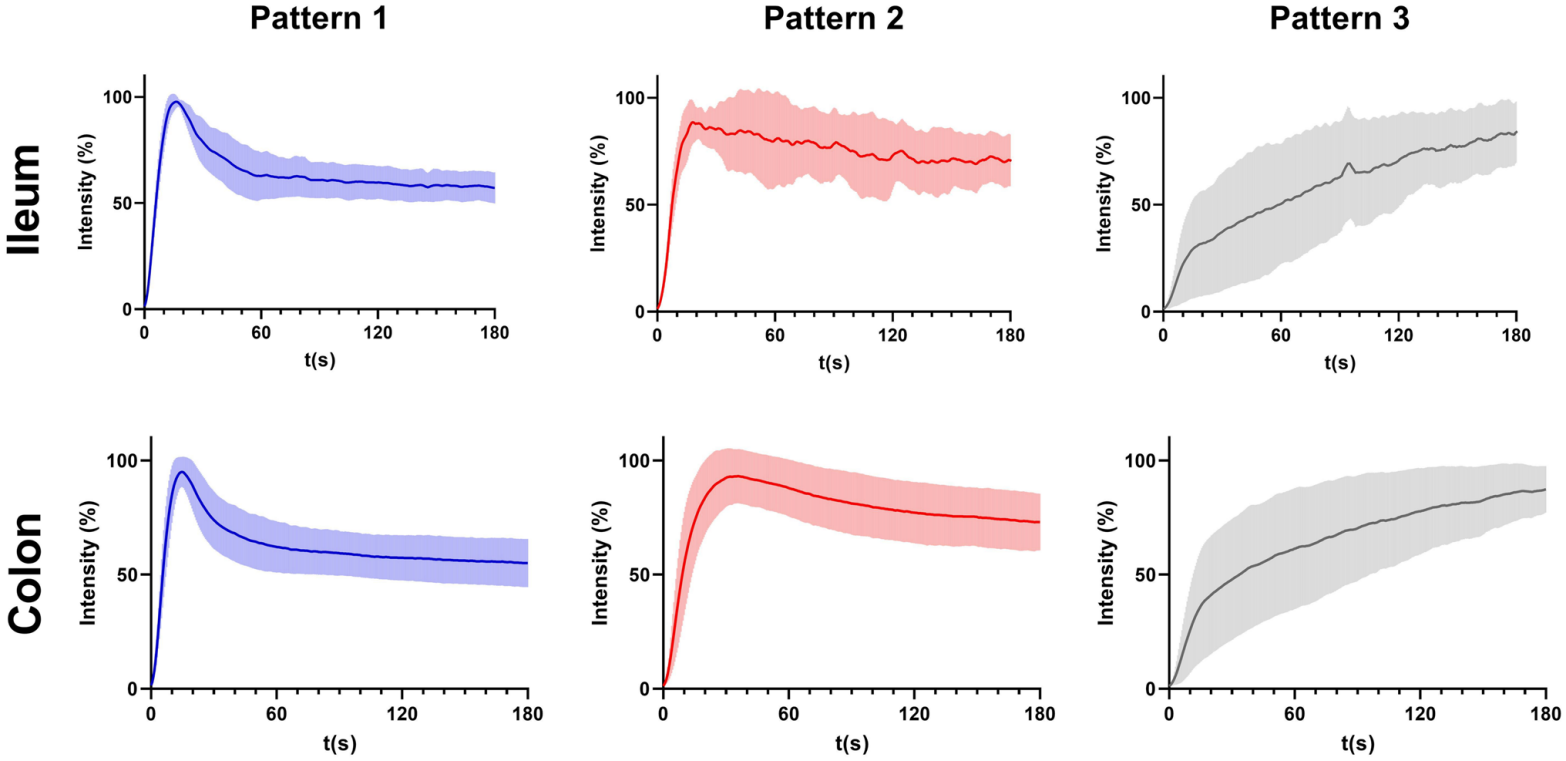
The mean normalized time-intensity curve with standard deviation of each perfusion pattern for both the ileum and colon

The time-intensity curves of the colon were distributed using cut-off values such as pattern 1: Tmax < 70 and Egress slope < − 1; pattern 2: Tmax < 70 and Egress slope ≥ − 1; pattern 3: Tmax ≥ 70. The mean normalized time-intensity curve with standard deviation of each perfusion pattern for both the ileum and colon are shown in Fig. 2.

The mean quantified perfusion parameters of each perfusion pattern were compared for both the ileum and colon. The Imax, Ingress slope, Ingress rate, Normalized slope, Tmax, and Egress slope of the ileum differed significantly between the three perfusion patterns. The AUC30, 60, 120 and 180 did not differ significantly. The perfusion parameters of the colon were all significantly different between the three perfusion patterns as shown in Table 2.

**Table 2:** In- and outflow parameters of the quantified time-intensity curves of the ileum and colon

Ileum	Pattern 1 (*n* = 17)	Pattern 2 (*n* = 8)	Pattern 3 (*n* = 10)	*p* value
Imax, a.u. (range)	131.71 (70.07–162.21)	48.75 (13.32–64.43)	39.37 (4.15–77.85)	** < 0.001**
Ingress slope, a.u./s (range)	15.45 (4.79–17.60)	5.46 (1.55–7.41)	1.18 (0.45–9.87)	** < 0.001**
Ingress rate, a.u./s (range)	8.59 (2.49–10.17)	1.14 (0.69–2.87)	0.16 (0.02–0.56)	** < 0.001**
Normalized slope, %/s (range)	11.99 (8.36–14.90)	9.90 (5.75–15.22)	4.66 (1.59–20.98)	**0.018**
Tmax, s (range)	14.50 (12.00–25.50)	27.00 (13.00–60.00)	215.25 (84.00–272.50)	** < 0.001**
Egress slope, a.u./s (range)	-4.18 (-8.59 to -2.51)	-1.26 (-2.31 to -0.58)	-0.54 (-8.42 to -0.06)	** < 0.001**
AUC 30, % (range)	78.12 (71.96–94.51)	95.59 (68.39–96.99)	96.46 (49.01–97.23)	0.076
AUC 60, % (range)	68.39 (62.21–86.28)	90.23 (57.68–95.86)	96.84 (49.02–96.92)	0.135
AUC 120, % (range)	62.14 (57.53–82.08)	82.11 (53.45–92.56)	95.91 (49.83–96.74)	0.191
AUC 180, % (range)	60.12 (55.38–79.87)	77.98 (52.38–91.61)	94.07 (94.07–94.07)	0.067

Colon	Pattern 1 (*n* = 95)	Pattern 2 (*n* = 32)	Pattern 3 (*n* = 56)	*p* value
Imax, a.u. (range)	87.49 (30.83–193.32)	46.76 (2.61–88.61)	25.22 (2.37–86.59)	** < 0.001**
Ingress slope, a.u./sec (range)	9.86 (3.73–35.03)	3.41 (0.22–11.29)	0.70 (0.11–10.98)	** < 0.001**
Ingress rate, a.u./sec (range)	5.51 (1.30–21.06)	1.21 (0.12–5.12)	0.10 (0.01–0.62)	** < 0.001**
Normalized slope, %/sec (range)	12.18 (6.58–19.38)	8.16 (3.80–18.39)	4.16 (1.43–12.68)	** < 0.001**
Tmax, sec (range)	13.00 (8.00–39.50)	29.75 (8.00–63.00)	231.00 (82.00–270.00)	** < 0.001**
Egress slope, a.u./sec (range)	-2.44 (-12.06 to -1.04)	-0.59 (-0.97 to -0.14)	-0.26 (-4.30 to -0.00)	** < 0.001**
AUC 30, % (range)	77.56 (59.59–94.85)	94.82 (52.69–98.08)	96.91 (78.93–99.28)	** < 0.001**
AUC 60, % (range)	68.44 (52.78–91.08)	89.52 (42.54–96.91)	95.80 (80.53–98.11)	** < 0.001**
AUC 120, % (range)	61.85 (48.08–95.90)	84.47 (38.40–96.70)	93.35 (81.37–96.11)	** < 0.001**
AUC 180, % (range)	58.43 (45.96–85.49)	81.09 (37.95–95.01)	85.59 (81.32–93.49)	** < 0.001**

All significant *p*-values are shown in bold to highlight significant parameters relative to non-significant parameters

*White rows* inflow parameters; *Grey rows* outflow parameters; *a.u.* arbitrary units; *sec* second; *Fmax* maximum fluorescence intensity, *Ingress slope* maximum inflow slope, *Ingress rate* mean slope from baseline to maximum fluorescence intensity, *Normalized slope* maximum inflow slope in percentage per second, *Tmax* time to maximum intensity, *Egress slope* maximum outflow slope, *AUC30* area under de curve after 30 s, *AUC60* area under de curve after 60 s, *AUC120* area under de curve after 120 s, *AUC180* area under de curve after 180 s

### Subjective interpretation of ICG NIR fluorescence imaging

**Fig. 3:**
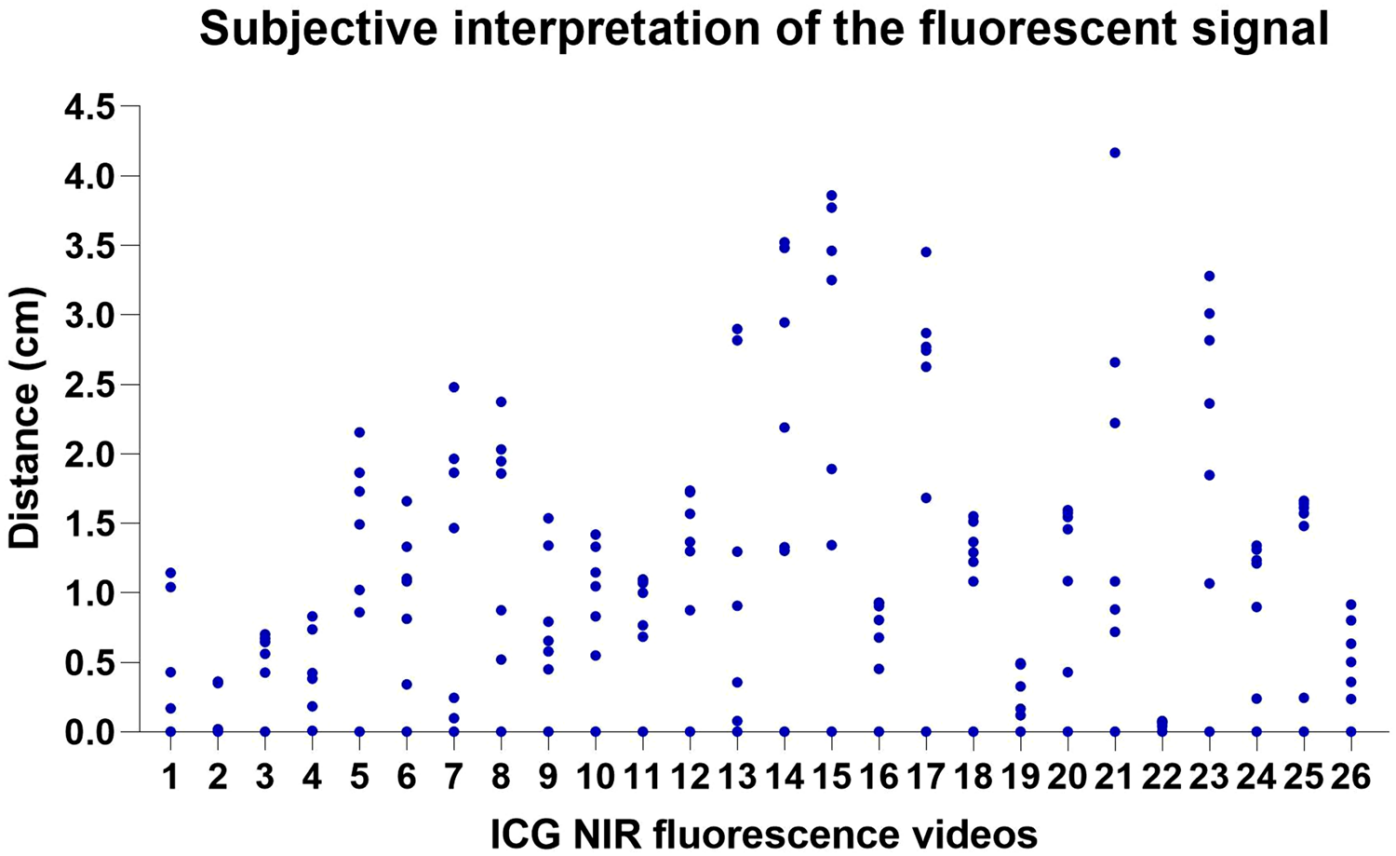
Inter-observer agreement of the surgeon’s subjective interpretation of the fluorescent signal per ICG NIR fluorescence video. Each *blue* dot represents an intended resection line marked by the surgeon based on the fluorescence signal, with the most proximal marker used as the baseline. An ICG NIR fluorescence video of both the afferent ileum or colon and the efferent colon was analyzed separately. Intraclass correlation (ICC) of 0.378 (95% CI 0.210–0.579)

All (*n* = 20) ICG NIR fluorescence imaging videos were independently assessed by 7 colorectal surgeons. The inter-observer agreement of the intended resection lines determined by the surgeons based on their subjective interpretation of the fluorescence signal was poor—moderate, with an ICC of 0.378 (95% CI 0.210–0.579). The median distance between the most proximal marker (i.e., baseline) and the most distal marker on the afferent and/or efferent ileum or colon was 1.122 cm (range 0.071–3.861). An overview of the distances (in centimeter) between the markers (i.e., intended resection lines) per fluorescence video are demonstrated in an ICC dot plot in Fig. 3.

**Fig. 4:**
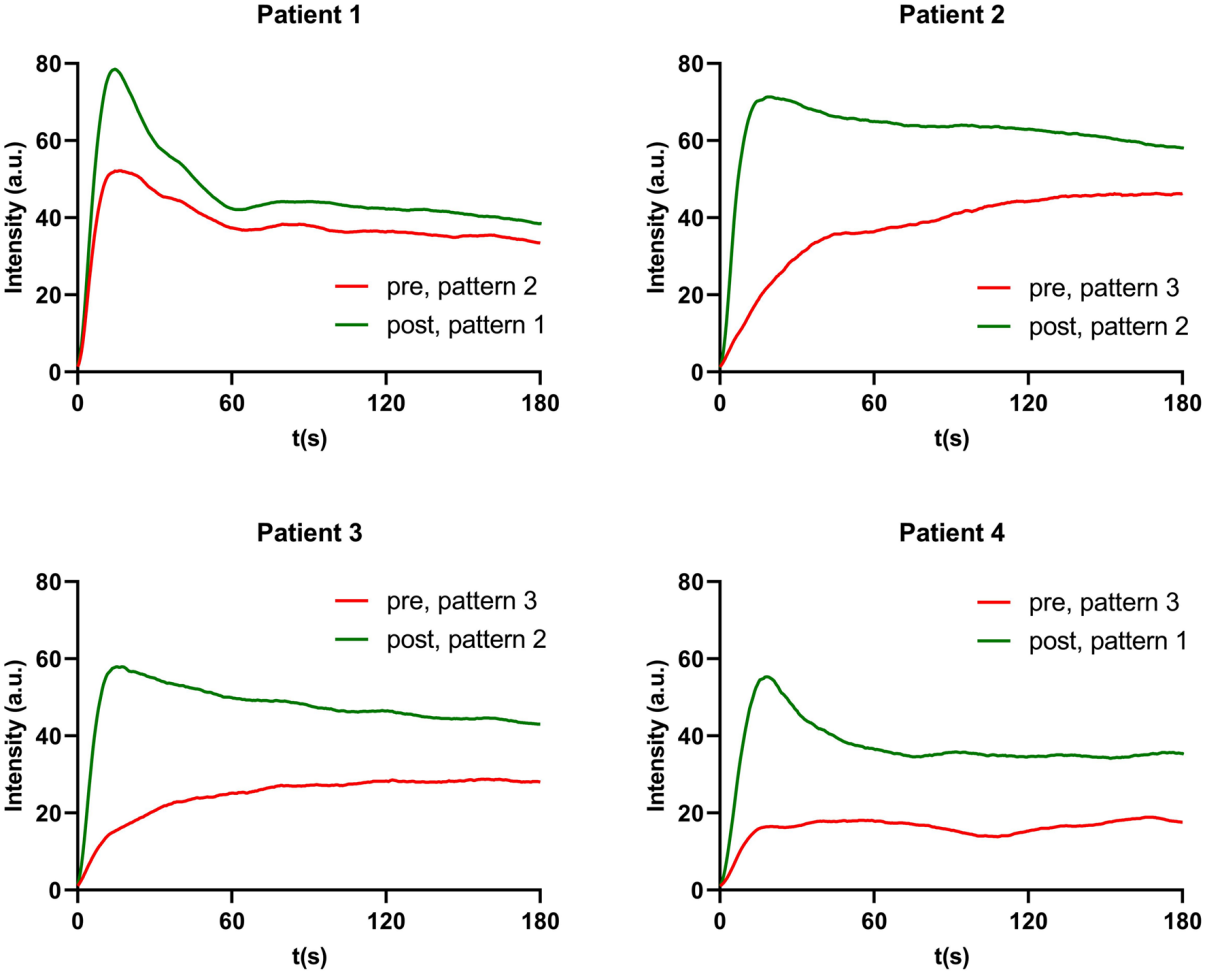
Time-intensity curves before and after additional bowel resection based on the surgeon’s subjective interpretation of the fluorescence signal in 4 patients. The *red* time-intensity curves represent the fluorescence signal of the intended anastomosis and the *green* time-intensity curves represent the fluorescence signal of the actual anastomosis after additional bowel resection (Color figure online)

The surgical plan has been changed in 4 (20%) patients by performing an additional bowel resection based on the intraoperative subjective interpretation of the fluorescence signal by the surgeon (Fig. 4). In 1 patient, perfusion pattern 2 was observed at the location of the intended anastomosis. After performing additional bowel resection, perfusion pattern 1 was observed.

Perfusion pattern 3 was observed in the other 3 patients at the location of the intended anastomosis and after additional bowel resection, perfusion pattern 2 was observed in 2 patients and perfusion pattern 1 in another patient. In none of these patients an AL occurred after 90 days of follow-up.

### Clinical outcome

**Fig. 5:**
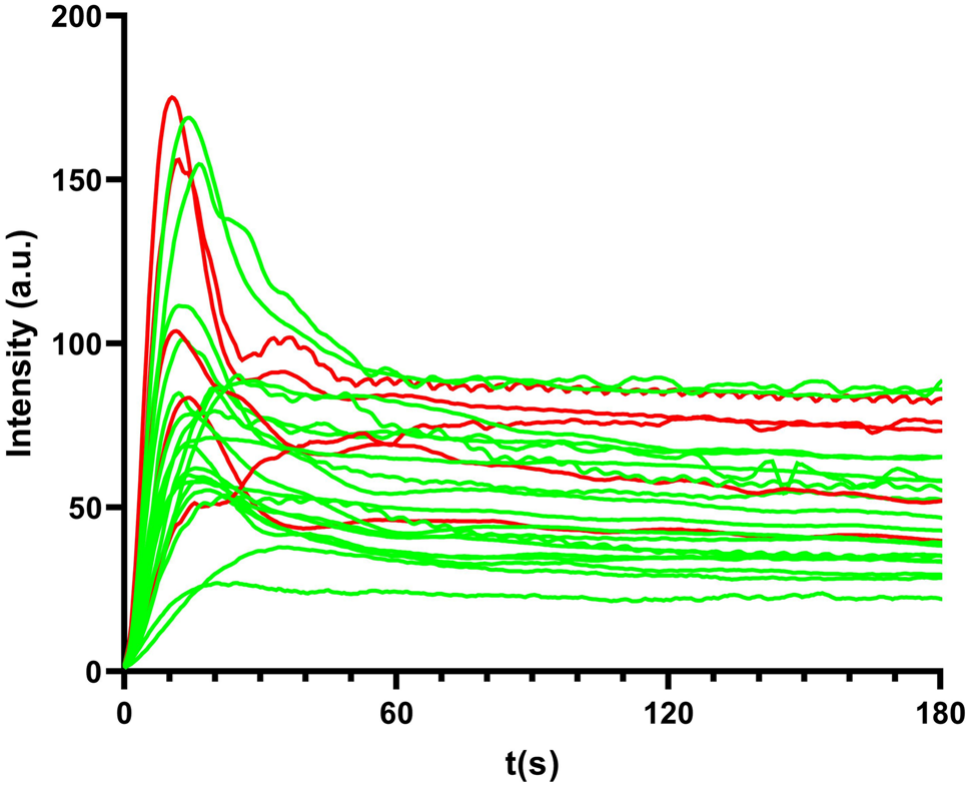
The absolute time-intensity curves plotted from the ROI of the anastomosis. The *red* absolute time-intensity curves represents the patients who developed an anastomotic leakage

A total of 24 time-intensity curves plotted from the ROIs of the anastomosis [afferent ileum or colon and efferent colon in 5 patients (*n* = 10); afferent ileum or colon alone in 14 patients (*n* = 14)] were analyzed (Fig. 5). Of all (*n* = 24) time-intensity curves, 17 (71%) corresponded to perfusion pattern 1, 6 (25%) time-intensity curves to perfusion pattern 2, and 1 (4%) time-intensity curve corresponded to perfusion pattern 3.

AL occurred in 4 (20%) patients. In 1 patient who underwent a right hemicolectomy, the time-intensity curve of the anastomosis on the afferent ileum corresponded to perfusion pattern 3 and the efferent colon to perfusion pattern 1. Postoperatively, this patient developed an acute coagulation disorder with subsequent bowel ischemia and anastomotic leakage as was observed during relaparotomy, after which the patient died. The other 3 patients who developed anastomotic leakage, underwent left-sided resection and the time-intensity curve of the anastomosis on the afferent colon corresponded to perfusion pattern 1. In 1 of these 3 patients, macroscopic ischemia on the afferent colon was observed during relaparotomy.

## Discussion

This prospective dual center cohort study identified quantified bowel perfusion patterns in patients undergoing colorectal surgery by using a standardized imaging protocol. A clear overview of the bowel perfusion was obtained by drawing contiguous ROIs from proximal to distal of the resection line on the ileum and afferent/efferent colon. The quantified time-intensity curves plotted from these ROIs could be divided into three different perfusion patterns. Perfusion pattern 1 implied for well-perfused bowel tissue, as it was mainly observed at the most proximal side of the resection line on the afferent ileum or colon and at the most distal side of the resection line on the efferent colon. In contrast, perfusion pattern 3 implied for poorly perfused bowel tissue, since it was generally present at the avascular region of the ileum or colon from which the vascular branch was already dissected. Perfusion pattern 2 implied for the transition zone, where perfusion pattern 1 transitions to perfusion pattern 3. These three perfusion patterns were identified in each patient and might be a representation of the actual bowel perfusion.

Several cohort studies have investigated quantified bowel perfusion using various quantification methods [14–25]. In contrast to our study, most of these studies did not use a standardized imaging protocol (i.e., inconsistency in camera-to-target distance, angle of camera on target tissue, type of imaging system and its settings, etc.). Moreover, the ROIs were selected based on the surgeon’s subjective interpretation of the fluorescence signal [14–16, 18–21, 23–25]. As a result, the study results were difficult to compare, which may negatively affect the reproducibility of previously reported quantification methods. Although in our study cut-off values to divide the time-intensity curves among the three perfusion patterns were determined based on qualitative assessment of the quantified time-intensity curves, the subjective factor was limited. Therefore, our quantification method might be more reproducible, which should be validated in larger studies.

Currently, ICG NIR fluorescence bowel perfusion assessment relies on the surgeon’s subjective interpretation of the fluorescence signal to guide clinical decision-making, which limits the reproducibility and validity of the technique [10–13]. This is underlined in this study where a poor-moderate inter-observer agreement of the surgeon’s subjective interpretation of the fluorescence signal was observed, with an ICC of 0.378 (95% CI 0.210–0.579).

The ICC was lower compared to the previous published study by Hardy et al. [12], in which a good ICC of 0.753 (95% IC 0.510–0.932) for experts and a moderate ICC of 0.613 (95% IC 0.409–0.856) for non-experts were found. In this study, there were some outliers in the surgeon’s subjective interpretation of the fluorescence signal (e.g., ICG NIR fluorescence videos 14, 15, and 21; Fig. 3) for which no clear explanation was found. A possible explanation for this variability could be the different ways in which surgeons interpret the fluorescence signal, with one surgeon focusing on eventually the most distal fluorescent resection line, another surgeon assessing the fluorescence signal by focusing on the time to maximum fluorescence, and other surgeons being more cautious in defining the resection line based on the fluorescence signal. Although a low ICC is not necessarily detrimental to the clinical outcome (i.e., AL), this study demonstrates the need for quantification of ICG NIR fluorescence imaging to improve the objectivity and accuracy of the bowel perfusion assessment. Especially when bowel length preservation becomes more important, for example in re-resections, Crohn’s disease, or in ultralow anterior resection.

The AL rate (20%) in this study was remarkably high when compared to the national AL rate of 6% in 2016 according to the Dutch Colorectal Audit (DCRA) [31]. This AL rate is believed to be an overestimation due to the small sample size as both participating hospitals have annual AL rates that are comparable with the national AL rate by the DCRA. Moreover, the high AL rate could also be explained by the fact that all included patients underwent surgery in tertiary academic hospitals that provide healthcare in highly complex patients in whom the a priori risk of developing AL will be higher. This could also be an explanation of the mortality rate of 10% (*n* = 2) in our study. One patient who developed AL was postoperatively admitted to the intensive care unit for respiratory insufficiency due to an aspiration pneumonia, followed by an acute coagulation disorder which resulted in an acute ischemic limb and bowel ischemia with AL as seen during relaparotomy, after which the patient died. The second patient (ASA-3 with cardiovascular risk factors such as smoking, obesity and diabetes mellitus 2) died of acute myocardial infarction postoperatively. In the other patients with AL, one patient underwent robotic low anterior resection (LAR) and a relaparotomy was performed three days postoperatively to evacuate a hematoma in the small pelvis.

A vital anastomosis was observed during this procedure, however, nine days after primary surgery, the patient developed AL without any signs of ischemia on the afferent colon during relaparotomy. In another patient using long-term prednisolone for IgG4 cholangiopathy, an AL with macroscopic ischemia on the afferent colon was observed. The last patient developed AL in whom LAR was combined with a left nephrectomy. During relaparotomy, a limited AL was observed that required a diverting stoma. Given these highly complex cases, no firm conclusions can be drawn about the high AL and mortality rates found in this study.

Although this pilot study was too small to assess the correlation between the quantified perfusion patterns and AL, the perfusion patterns of the patients who developed AL (*n* = 4) were analyzed. Interestingly, in three of these patients the time-intensity curves of the anastomosis corresponded to perfusion pattern 1. However, in only one of them macroscopic ischemia on the afferent colon was observed during relaparotomy. This may be explained by the fact that ICG NIR fluorescence bowel perfusion assessment has been implemented as standard care, allowing the surgeon to change the surgical plan intraoperatively based on subjective interpretation of the fluorescence signal. As a result, risk factors other than tissue perfusion might be overrepresented in this cohort, since the development of AL is known to have a multifactorial cause. Future studies should investigate the predictive value of each perfusion pattern on the development of AL.

This study has some limitations. First, the primary goal of this exploratory study was to identify quantified bowel perfusion patterns. Therefore, the study design does not allow for any firm conclusions to be drawn about the reliability of the determined cut-off values distributing the time-intensity curves among the three perfusion patterns and the correlation between each perfusion pattern and the development of AL. Thus, there might be a risk of random sampling errors. Additionally, variation in patient-specific hemodynamic factors or the use of vasopressors during the ICG NIR fluorescence measurement could affect the observed perfusion pattern which requires further evaluation because of limited evidence in literature [32]. Second, our standardized imaging protocol only allows for extracorporeal video recordings of the ICG NIR fluorescence bowel perfusion assessment. Therefore, in some patients undergoing laparoscopic or robotic left-sided resection, only quantification of the ICG NIR fluorescence imaging video of the afferent colon was possible.

Standardization of the camera-to-tissue distance and angle of the camera-to-tissue can be challenging to maintain during intra-abdominal imaging. However, some studies have shown that quantification of the fluorescence signal using a laparoscopic imaging system might be possible, but the technique of this intracorporeal method is still in its early stages and needs further optimization [23, 33].

Lastly, even though the quantification tool is suited to produce proper time-intensity curves in most patients (*n* = 18), the motion tracker of the quantification tool was unable to correct for severe breathing-related movements in two patients. These movements resulted in a fluctuating line in the time-intensity curve, which affected the accuracy of some perfusion parameters (e.g., Tmax or Egress slope). Although this effect was negligible in our study, the quantification software needs further improvement for daily use.

In the future, larger studies with powered sample sizes should investigate the variation in perfusion patterns and corresponding cut-off values within various bowel parts in correlation to the occurrence of an AL. In addition, these studies should give us a conclusive answer whether quantified perfusion patterns are a reflection of the actual bowel perfusion status and if these pattern could be of added value to predict AL intraoperatively. Moreover, quantification of ICG NIR fluorescence bowel perfusion assessment should be performed intraoperatively to guide the surgeon’s clinical decision-making during surgery and to allow immediate modification of the surgical plan when needed.

## Conclusion

In conclusion, this prospective cohort study showed that quantification of ICG NIR fluorescence bowel perfusion assessment is a feasible method to differentiate between different perfusion patterns. The use of a standardized imaging protocol could improve the reproducibility of the quantification method. Moreover, the poor-moderate inter-observer agreement of the subjective interpretation of the fluorescence signal between surgeons emphasizes the need for quantification of the fluorescent signal to improve the objectivity and accuracy of the bowel perfusion assessment. Future studies should examine the clinical value of these different perfusion patterns by correlating each perfusion pattern with the development of AL.

## Supplementary Information

Below is the link to the electronic supplementary material.

Supplementary video 1. An example of a standardized fluorescence measurement (MP4 26051 kb)
